# Development, fabrication and mechanical characterisation of auxetic bicycle handlebar grip

**DOI:** 10.1038/s41598-023-35418-8

**Published:** 2023-05-19

**Authors:** Nejc Novak, Vasja Plesec, Gregor Harih, Andrej Cupar, Jasmin Kaljun, Matej Vesenjak

**Affiliations:** Faculty of Mechanical Engineering, University of Maribor, Maribor, Slovenia

**Keywords:** Mechanical engineering, Polymers

## Abstract

The auxetic cellular structures are one of the most promising metamaterials for vibration damping and crash absorption applications. Therefore, their use in the bicycle handlebar grip was studied in this work. A preliminary computational design study was performed using various auxetic and non-auxetic geometries under four load cases, which can typically appear. The most representative geometries were then selected and fabricated using additive manufacturing. These geometries were then experimentally tested to validate the discrete and homogenised computational models. The homogenised computational model was then used to analyse the biomechanical behaviour of the handlebar grip. It was observed that handle grip made from auxetic cellular metamaterials reduce the high contact pressures, provide similar stability and hereby improve the handlebar ergonomics.

## Introduction

Cellular structures are made of an interconnected network of solid struts or sheets which form the cell’s edges and faces. Their mechanical behaviour depends mainly on the relative density (porosity) and the base material, which can be metal or non-metal^1^. Recently, a special kind of cellular structures (metamaterials) was introduced that exhibits an auxetic behaviour under mechanical loading (auxetic metamaterials)^2^, characterised by negative Poisson’s ratio (NPR), resulting in a counterintuitive behaviour when subjected to mechanical loading^3,4^. Auxetic metamaterials tend to expand in the lateral direction in the case of longitudinal tensile loading and vice versa in the case of compressive loading. Such behaviour is made possible by a two- or three-dimensional hinge-like skeleton with nano, micro or macro predefined and optimised arbitrary geometry that acts like a mechanism by unhinging crumbled cells or rotation of rigid units^4^. Some auxetic materials might also be found in nature, i.e. rocks, minerals, crystals, bone tissue^4^. In some cases, the NPR significantly enhances mechanical properties, which is useful for many applications in engineering, medicine, fashion and sports^4,5^. The auxetic materials have an improved shear performance (increased shear modulus)^2^, mechanical vibration damping, sound^6^ (also with forbidden band control^7^) and energy absorption^8–10^ capabilities and allow for predefinition of the crack propagation path^11,12^. These properties make them particularly attractive and useful in modern applications as multifunctional materials since they can improve properties in many fields simultaneously. They are being increasingly studied for use in body and vehicle armour applications as cores in sandwich panels for ballistic protection^13,14^. Additionally, they can be used in sports as flexible pads to offer additional protection and to reduce the impact severity for various human body parts (e.g. elbow, leg, torso)^15,16^. The cell shape changes of auxetic metamaterials efficiently absorb energy since these changes rapidly spread through the complete volume of the auxetic metamaterial. Therefore, an impact on a small part of the auxetic structure will result in energy dissipation through the entire structure. This can be beneficial also for the bicycle handlebars. During bicycle riding, various loading cases on the hands occur, such as pushing, pulling, twisting, etc. Therefore, the bicyclist must firmly grasp the handlebar grip for safe riding. Previous research has shown a strong link between high grasping forces and hence loads on the hand tissue and the development of various cumulative trauma disorders^17–19^. Soft tissue (skin, subcutaneous tissue, muscle, fascia, etc.) shows highly non-linear mechanical behaviour with low stiffness at small strains with an increase of stiffness with larger strains. Several authors argued this is the main factor leading to sudden discomfort and pain during high-force grasping^20–22^. In this regard, handlebars need to be designed ergonomically to fit the user in terms of size and shape^23–27^. On the other hand, handlebar material choice has been mostly overlooked. Recent research has also shown that material choice significantly influences the biomechanical system hand-handle and hence handle ergonomics^28–31^. It has been indicated that ergonomics can be improved even further with deformable materials, which reduce the maximum contact pressures occurring during grasping with slight deformation of the handle material^28^. Results and measurements have shown that a carefully designed deformable handle can provide a higher comfort rating while maintaining stability^30^. In this manner rough guidelines for pressure discomfort (PDT) and pressure-pain threshold (PPT) were provided in the past. PPT is higher than PDT and values differ by the area of the hand and between different subjects. The suggested PDT limit is 188 kPa according^32^, while^33^ estimated the value to be 104 kPa. To maintain desired user performance, product handles should avoid shapes that result in high contact pressure during grasping. Designers must ensure handheld products distribute contact pressure evenly and do not exceed the PDT or PPT limits.

The auxetic and non-auxetic bicycle handlebars were designed, fabricated and analysed in this work in terms of mechanical and deformation behaviour. First, the appropriate geometries were selected with a preliminary parametric computational design study. The 3 different geometries were then fabricated and experimentally tested, followed by the validation of homogenised and discrete computational models. The homogenised computational models were then further used for the simulations of contact pressure distribution, deformations and displacements to take into account biomechanics and ergonomics.

## Parametric design study

The preliminary computational parametric design study was performed to determine the most suitable geometries for later fabrication and detailed mechanical characterisation.

### Geometry

The geometry of specimens was selected based on the fabricability with used fabrication technique and obtained using 3D software Rhinoceros 7 together with add-in Grasshopper. The main geometry values were the inner and outer diameter and height of the specimen. The specimen has been divided into segments in radial and circumferential directions. Geometrical models have been defined as surface models used in Finite Element Modelling (FEM) analyses and as solid models used for layered additive manufacturing of the physical specimens.

**Figure 1:**
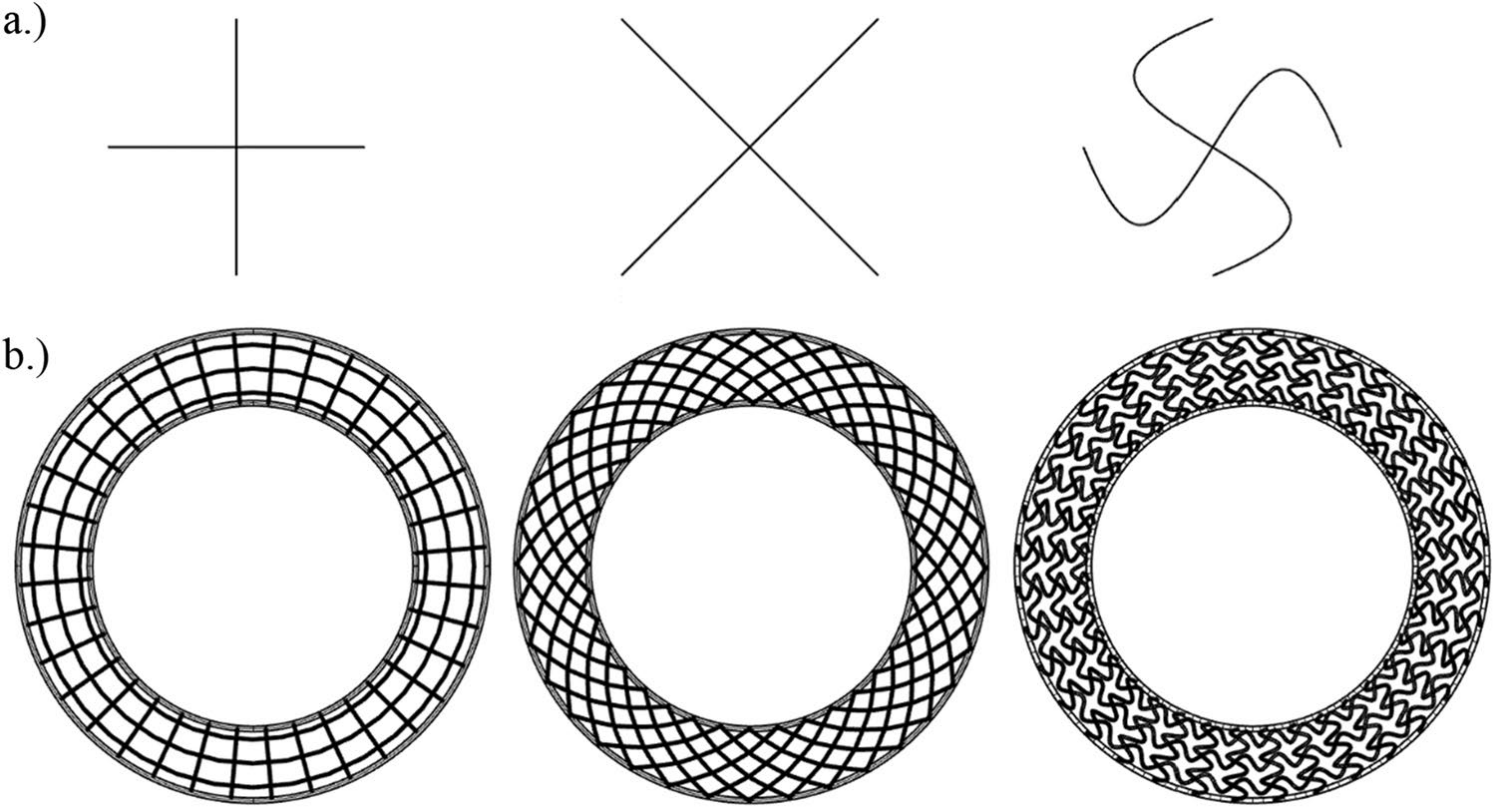
Basic unit cell structure shapes in cross-section: +, X, and S—(**a**) and models of three different infill shapes (**b**).

Three different basic unit cell shapes were chosen: + (non-auxetic), X (non-auxetic) and S (auxetic based on tetra chiral geometry, named also S-shape^34^), as shown in Fig. 1a. The geometry of S-shape is based on cubic Bezier curve defined by 4 control points.

**Figure 2:**
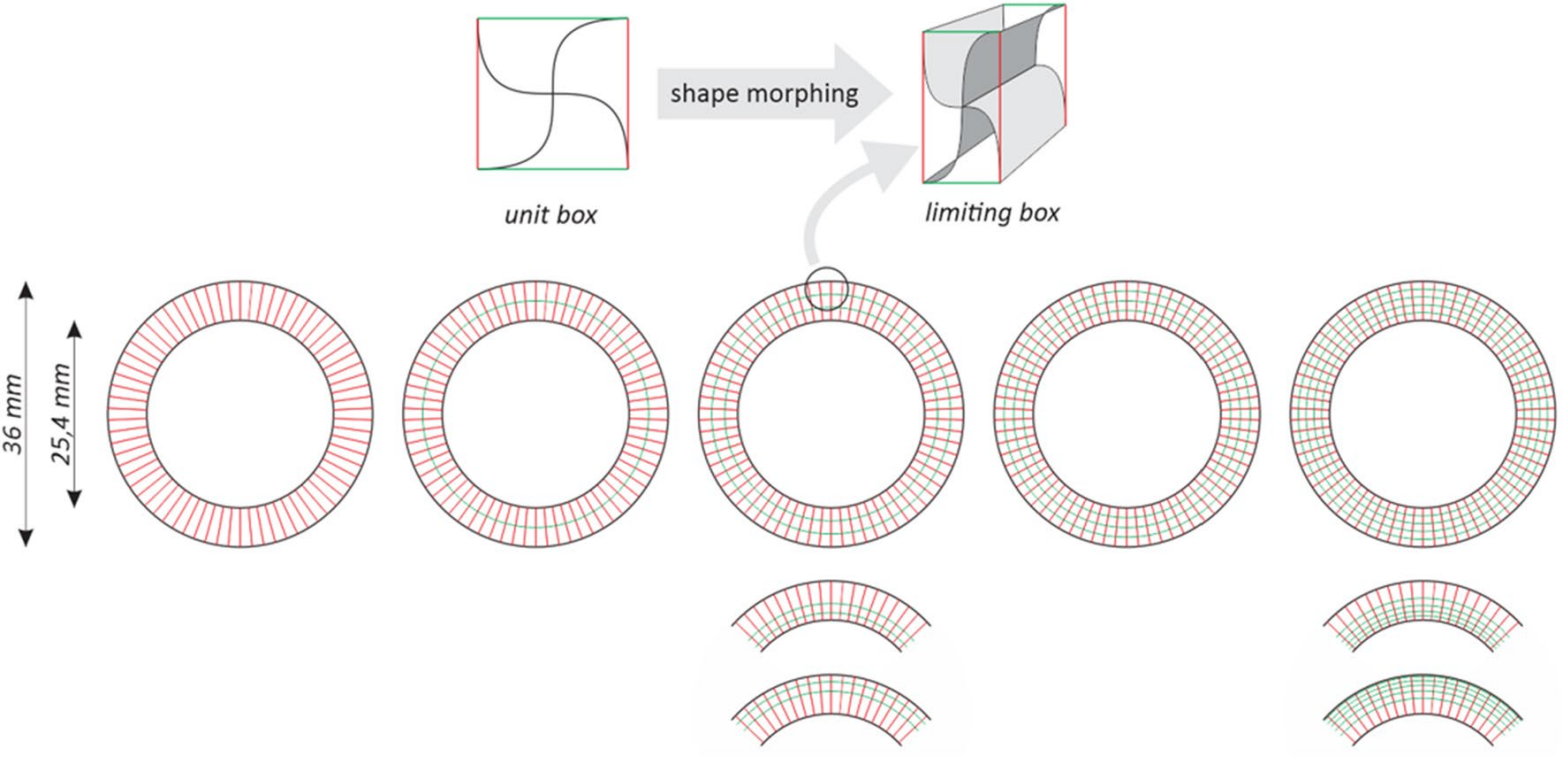
Boundary surfaces, limiting boxes, radial gradation.

Initial cylindrical shapes (inner and outer wall of the grip) were used as a boundary for radial and circumferential distribution of limiting boxes (Fig. 2).

**Figure 3:**
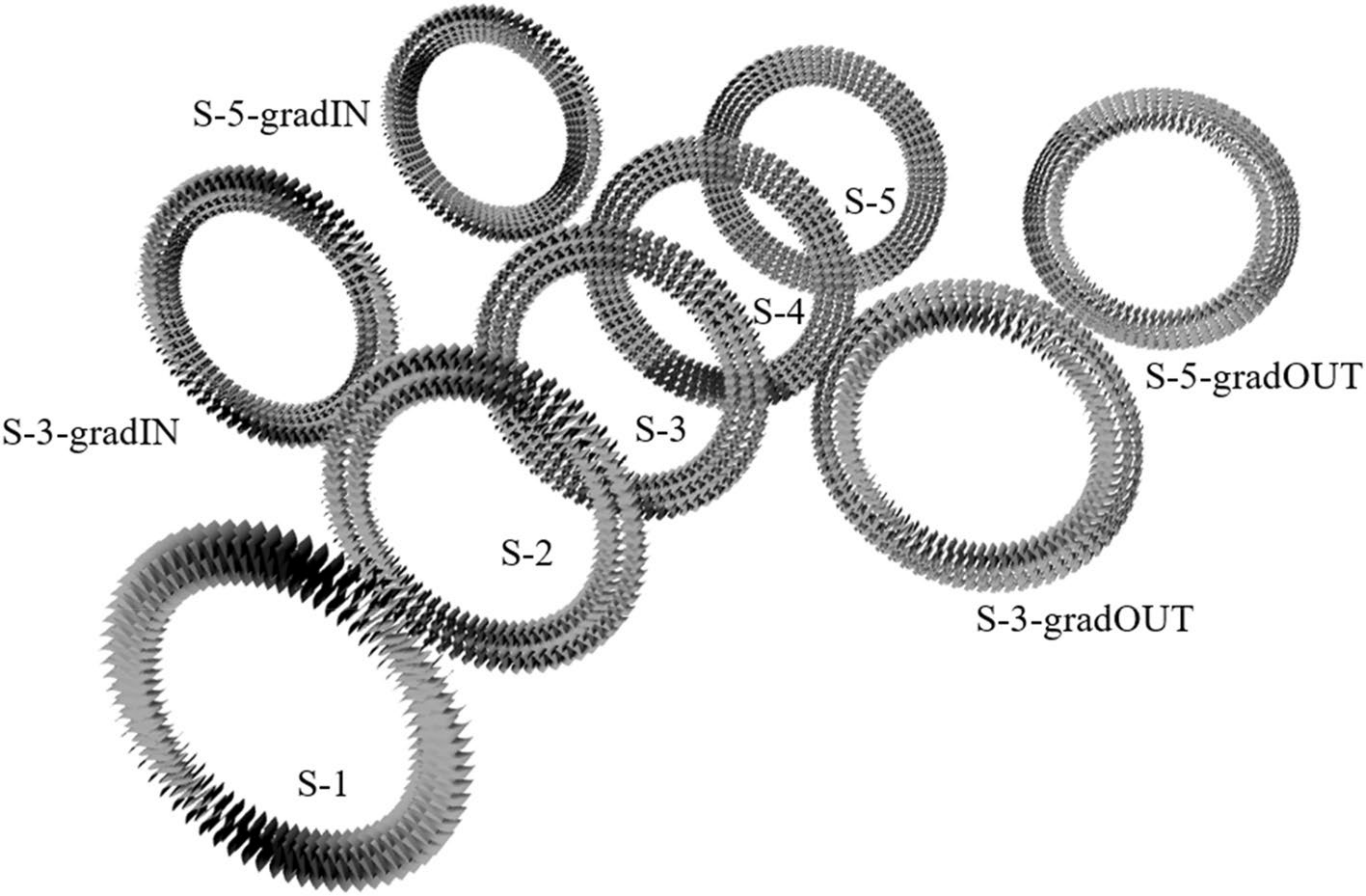
Set of geometries for S unit cell structure for preliminary parametric study.

Grasshopper procedure has been developed to control the radial and circumferential distribution of limiting boxes. To create surface models, “Twisted Box” and “Morph Box” functions were used. Unit cells have been morphed to limiting boxes using unit cells boundary box (cube). In contact areas, C0 (position continuity) has been defined. In the last step, individual cells have been joined to form one poly surface. The circumferential array has been set to 70 cells, while the radial array varied from 1 to 5 cells with equal and graded distribution (to outer and inner cylinder) as presented in Table 1. In that way, 9 geometries were obtained for each basic unit cell (Fig. 3). Each model has been exported to FEM environment using Step file format.

**Table 1:** Radial cell distribution—radial cell diameters.

	Inner diameter	Segments diameters (green lines)				Outer diameter
2 Segments	25.4	30.7				36
3 Segments	25.4	28.93	32.47			36
3 Segments grade IN	25.4	27.34	29.88			36
3 Segments grade OUT	25.4	31.52	34.06			36
4 Segments	25.4	28.05	30.7	33.35		36
5 Segments	25.4	27.52	29.64	31.76	33.88	36
5 Segments grade IN	25.4	26.54	27.82	29.34	31.32	36
5 Segments grade OUT	25.4	30.08	32.06	33.58	34.86	36

To produce virtually developed models, the 3D printer Tevo Tarantula was used. Initially, it was equipped with 0.4 mm nozzle but was replaced with 0.2 mm nozzle to achieve one-walled structure in 3D printed part. 3D printing material was TPU Flex from producer Azurefilm with 89A Shore hardness. Printing slicer used for this task was CURA 4.10.0 with the following printing parameters: T (nozzle): 228 °C, flow: 100%, printing velocity: 40 mm/s, no retraction, no supports, special mode: Surface Mode set to Surface. In one printing task, all three models were printed.

The specimens were fabricated with an inner diameter of 25.4 mm and an outer diameter of 36 mm and a height of 10 mm. Some adoptions of the specimen’s geometries, e.g. thickening inner and outer cylinder and reducing circumferential array to 35 cells, were needed for successful fabrication. Therefore, outer and inner walls were defined according to 3D printer’s nozzle diameter, which was 0.2 mm. This means that the printer nozzle draws only one line to create a wall. Between those shells, different inner structures were constructed (extruded) with a constant cross-section in height (z axis). In xy plane the structure was radial and circumferential arrayed using one basic cell shape placed in box. Above mentioned Grassopper procedure has been adopted to create the initial surface models. Three rows in radial direction and 35 segments in circumferential direction have been defined to assure the manufacturability of physical specimens. Created surfaces have been symmetrically offset at a total distance of 0.2 mm (Fig. 1b).

### Simulations

**Figure 4:**
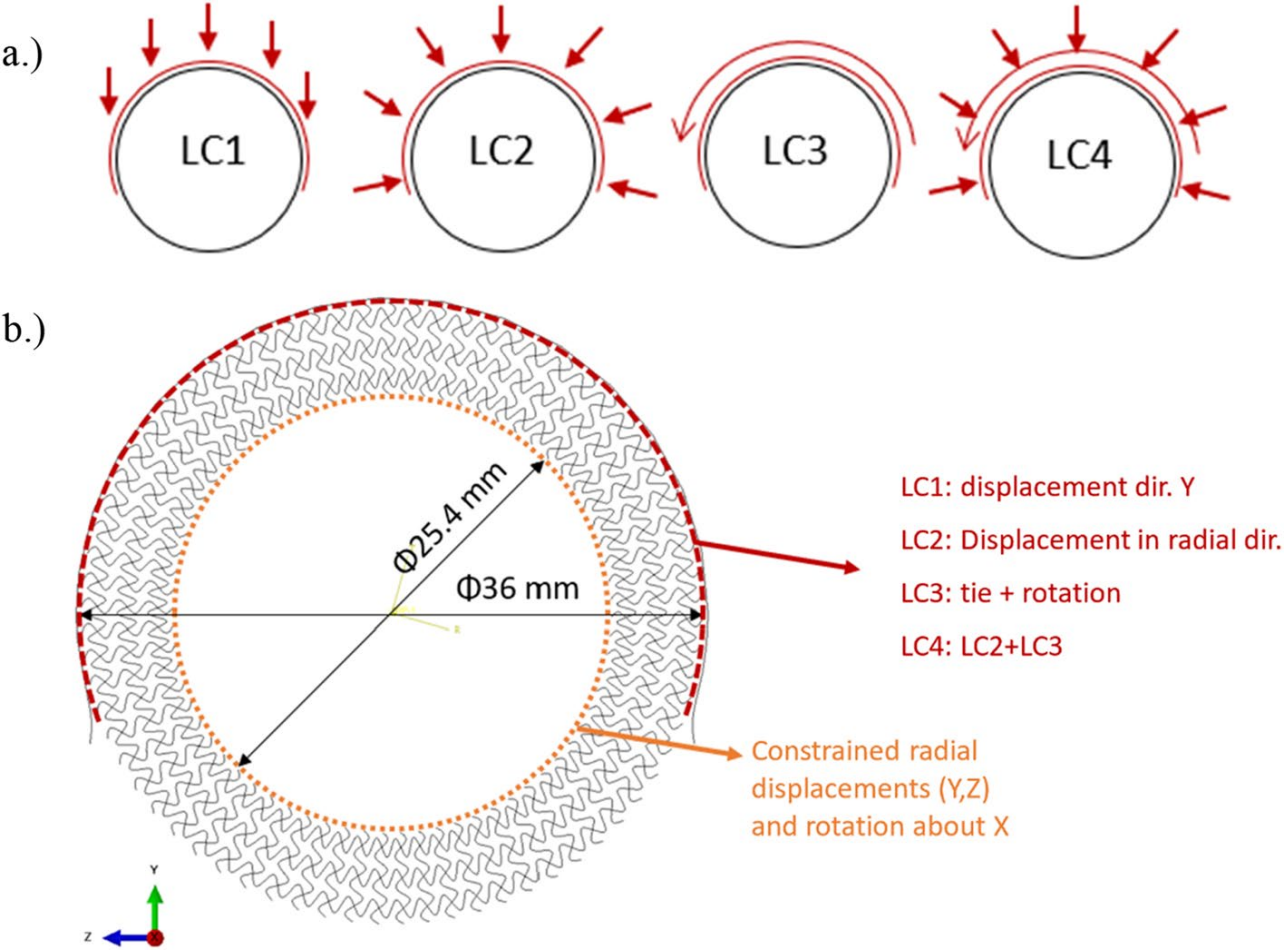
Load cases (**a**) and boundary conditions (**b**) of preliminary computational study.

All the simulations were performed in the finite element (FE) software Ansys, where 4 different load cases (LC) were taken into account, as shown in Fig. 4a. The prescribed boundary conditions are presented in Fig. 4b. The LCs were determined by the expected load on the handlebar, where LC1 represents load in radial direction (leaning/pressing on the handlebar), LC2 grabbing of the handlebar, LC3 rotation and LC4 grabbing and rotation of the handlebar.

Geometries are presented in Paragraph 2.1. All the analysed geometries in this preliminary study have the same relative density (the thickness of the shell finite elements were different for different geometries) and thus mass. They were discretised using the shell FE and generic bulk material properties described with linear elastic material model (density: 1250 kg/m^3^, Young’s modulus: 2500 MPa and Poisson’s ratio: 0). The global size of shell finite elements was 0.05 mm.

**Figure 5:**
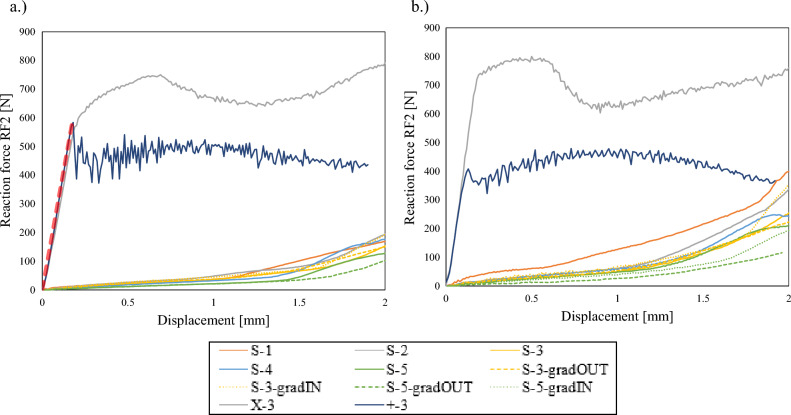
Reaction force of X, S and + structures in the case of LC1 (**a**) and LC2 (**b**).

**Figure 6:**
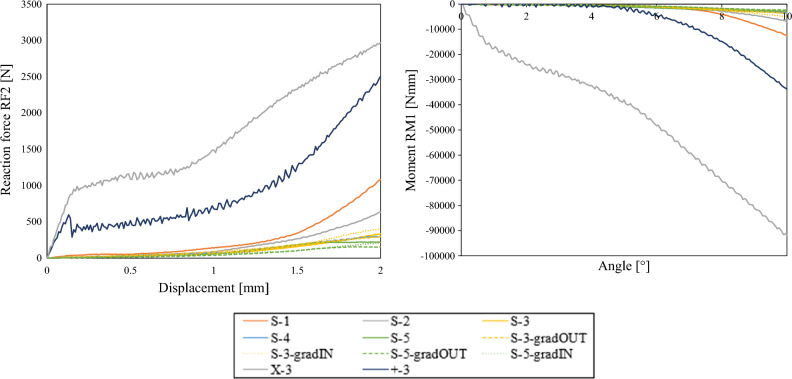
Reaction force and moment of X, S and + structures in the case of LC4.

The results of the preliminary computational study are shown in Figs. 5 and 6 for all analysed LCs, for each the most representative directional reaction force or/and moment. As can be seen from Fig. 5, the X and + geometries provide significantly stiffer responses in comparison to other analysed designs. This is a consequence of straight cell walls oriented in the radial direction, which are much stiffer than the already curved cell walls in the case of the S geometry. In general, the increase of the number of unit cells in the radial direction decreases the stiffness of the handlebar, which is evident from the zoomed area in Fig. 5. The gradation has different influence of the mechanical response in the case of 3 and 5 unit cells structures. While in the case of S-3, the gradation delays the stiffness increase, an opposite behaviour has been noted in the case of S-5. The gradIN and gradOUT geometries provide the same response with 5 unit cells in radial direction, while the gradIN geometry provides a stiffer response compared to gradOUT in the case of geometry with 3 unit cells in the radial direction.

In the case of LC2, the X and + provide the stiffest response again. The stiffness decreases with increasing the unit cell number in the radial direction in the case of S structures (Fig. 5).

In the case of LC3 and LC4, the mechanical responses are very similar. Therefore, only results of LC4 will be presented, where in addition to the reaction force also, the reaction moments around the x-axis are presented in Fig. 6. The X and + geometries are still the stiffest compared to S geometries, in which the stiffness decreases with increasing unit cell number.

**Figure 7:**
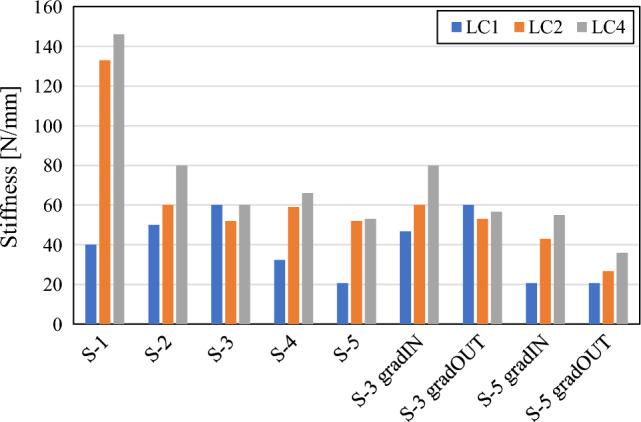
Stiffness comparison of X, S and + structures.

In order to compare the responses more precisely, the stiffness of the responses was evaluated as shown with red dashed line in Fig. 5a. The results are given in the Fig. 7, where the general trend follows the beforementioned conclusion as that in general stiffness decrease with increasing cell numbers. The stiffnesses of + and X configurations are in range of 3000 N/mm in case of LC1 to 5500 N/mm in case of LS 4, therefore they are not shown in the Fig. 7.

As can be seen from the results shown in Figs. 5, 6 and 7, the results differ a lot between the +, X and S structures, while the different geometry based on S unit cell don’t differ much, also when introducing the graded porosity. Therefore, the +, X and S-3 geometries were selected for subsequent fabrication experimental testing.

## Experimental testing

### Methods

**Figure 8:**
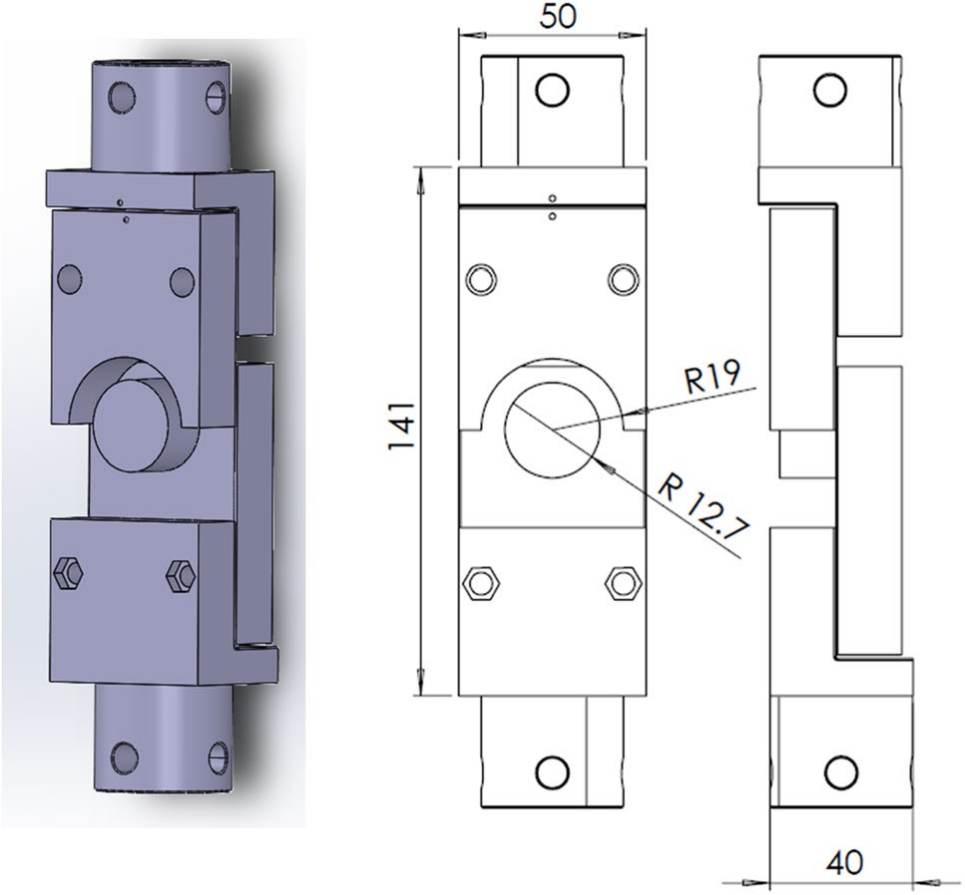
3D model and drawing of the clamping device.

The experimental tests were performed using the Tinius Olsen H10KT testing machine. The LC1 was chosen since the execution of the experiments is most straightforward. The clamps mimicking the LC1 (Fig. 4) were fabricated using additive manufacturing from PLA on FlashForge Creator 3 (Fig. 8). The 50 N load cell was used, and the loading velocity was set to 1 mm/min. Reaction force and displacement were monitored during the experimental testing. A high-definition camera has been used to follow the deformation behaviour.

### Ethical approval

The authors declare that they have no conflict of interest and no living organisms were harmed.

### Results

**Figure 9:**
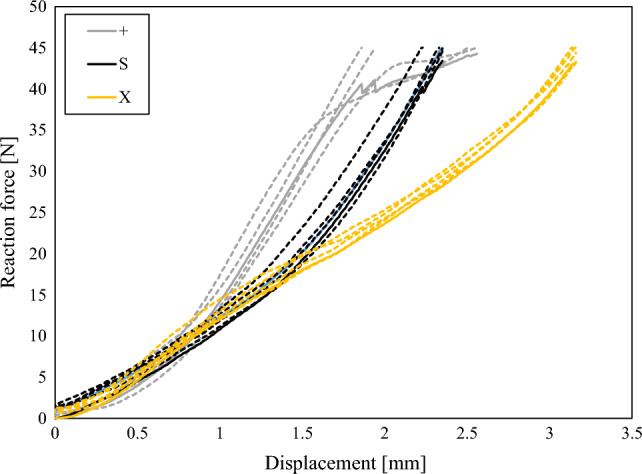
Experimental results of tested +, S and X structures with average responses shown with solid lines.

The mechanical responses of all samples are shown in Fig. 9, where the average responses are represented with solid lines. As can be observed, the initial responses up to 0.7 mm are very similar for all analysed geometries, while at larger displacements, the + geometry provides a much stiffer response than the others. This is a consequence of cell walls aligned with the radial direction. At displacements above 1.5 mm, the S geometry starts to densify, providing a stiffer response than X structure. Contribution to that stiffness difference is also by the 9% lower mass of the fabricated X geometry samples, which is a consequence of the same nozzle size used for the fabrication of all samples, resulting in the same wall thickness.

**Figure 10:**
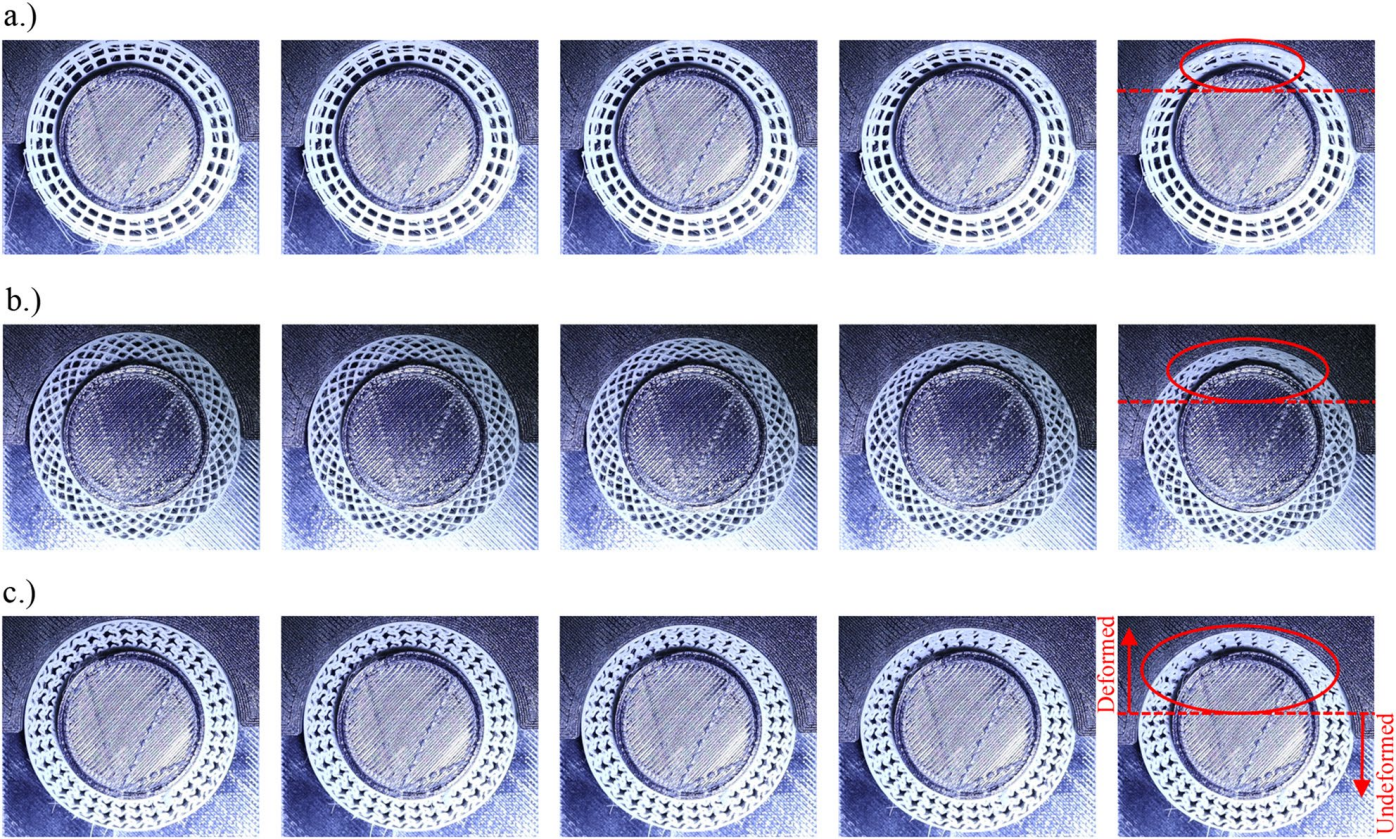
Deformation response of + (**a**), X (**b**) and S (**c**) structures taken at the same displacement values (displacement step: 0.5 mm) and with red ellipse and dashed line marked area with the deformation localisation.

The deformation response of all analysed structures is shown in Fig. 10. As can be seen from the marked areas on final deformation shape images, the S geometry deforms in a much wider area compared to other geometries. Besides this, also the deformation at the opposite side of loading is limited in S geometry, while the offset between the samples and clamps is observed at larger displacements in case of + and X geometries. Therefore, it can be concluded that it also contributes to the stiffness increase compared to the X geometry.

## Computational simulations

The results of discrete and homogenised computational models are shown in this section. The homogenised computational model offers a fast estimation of the cellular structure’s response, where the geometry is modelled with solid finite elements and not in a discrete and exact way of describing the geometry with the shell or beam finite elements. The global size of the volume finite elements was determined by the parametric study and set to 0.5 mm. The boundary conditions were the same for both analysed computational models (LC1), as shown in Fig. 4. The geometry of the discrete computational model was modelled in the same way for the discrete computational model as in the preliminary study. The linear-elastic material model parameters for discrete model (Young’s modulus: 1800 MPa and Poisson’s ratio: 0) were determined using the reverse engineering method comparing the experimental and computational results. Same way were determined also the linear-elastic material model parameters for homogenised model (Young’s modulus: 0.55 MPa (X), 0.3 MPa (+), 0.35 MPa (S) and Poisson’s ratio: 0).

### Validation of discrete and homogenised computational model

**Figure 11:**
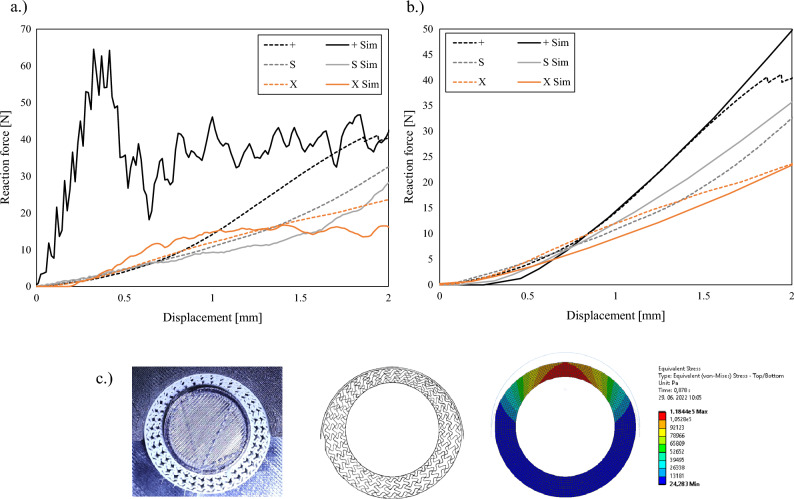
Comparison of experimental and computational results of discrete (**a**) and homogenised (**b**) models of +, S and X geometries and the deformation state of S structure (**c**) at displacement of 2 mm.

The comparison between the computational and experimental results is shown in Fig. 11. In the case of the discrete computational model (Fig. 11a), the good correlation can be observed for S and X geometry, while there is a large discrepancy in the case of + geometry in the initial stage of loading. This is a consequence of the very stiff out-of-plane behaviour of shell FE, where a much larger loads are needed to bend the cell wall as in the case of real structure made from TPU. In the case of + structure are some of the walls oriented directly in the radial direction and therefore several buckling of these walls is needed to deform the structure. In the case of X structure, none of the walls are oriented in the radial direction and are therefore easier to deform with the bending deformation mode. The correlation between the experimental and the computational results is good for all analysed geometries in the case of the homogenised computational model.

### 3D finger simulations

The commercial FE simulation software Ansys was used to simulate a finger grasping the developed cellular metamaterial grip. The results were compared to a quasi-rigid steel and conventional solid handlebar grip material-rubber. Results in terms of contact pressure distribution, deformations and displacements are analysed in terms of biomechanics and ergonomics in later subsections.

#### Biomechanical computational model

Fingertip bone was considered to be rigid, since its elastic modulus is magnitudes higher compared to skin and soft tissue. Skin and subcutaneous tissue exhibit non-linear behaviour, therefore a proven Ogden hyper-elastic material model was used based on data from previous study^35^.

Homogenised computational material model described above was used for the material of the auxetic cellular metamaterial handlebar grip. Additionally, steel as a quasi-rigid handlebar material and rubber as the most common material for bicycle handlebar grips were considered and defined as an isotropic elastic material with Young’s modulus of 2 × 10^5^ MPa and 33.7 MPa and a Poisson’s ratio of 0.3 and 0.4874, respectively, using the Ansys material database.

**Figure 12:**
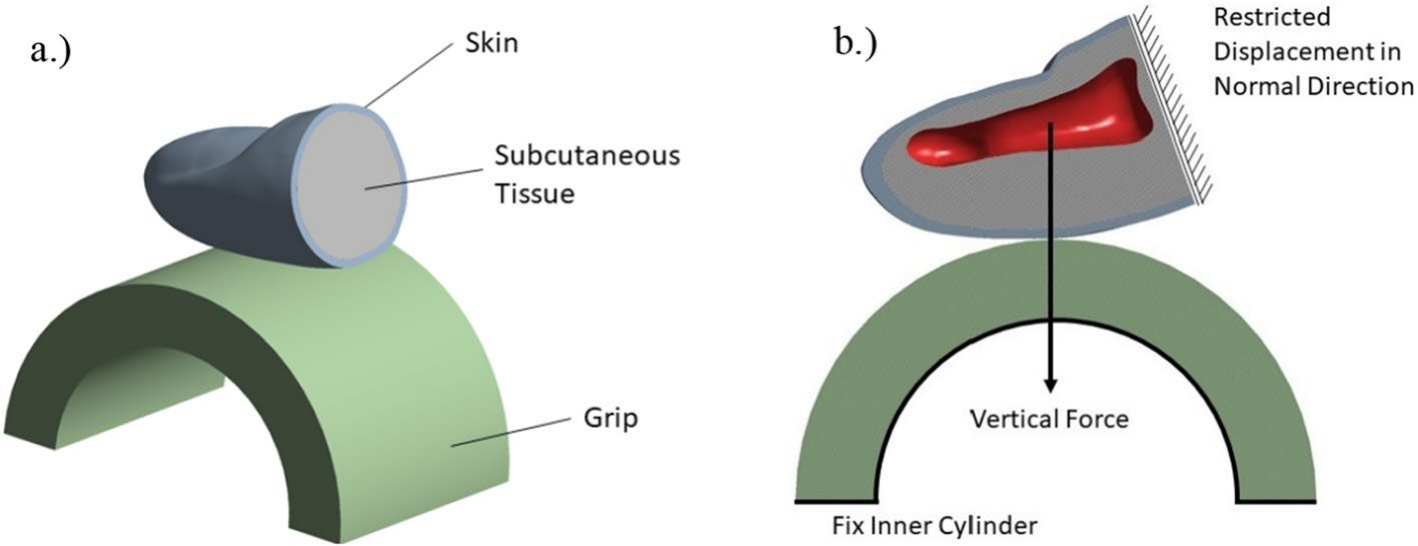
Geometry and material properties assignment of the model (**a**) and boundary conditions (**b**).

The geometry of the fingertip was obtained using medical imaging and reverse engineering technology. An average sized human finger according to anthropometric measurements was considered (Fig. 12a). The bicycle handlebar grip was modelled as half cylinder with outer diameter of 36 mm and proposed material thickness of 5.5 mm. The inner part of the cylinder was fixed (no translations and rotations). Finger was positioned according to normal power grasping scenario with resulting normal force on the distal phalange. Additionally, finger bone was fixed in all directions, except the direction of the vertical force. Hereby a numerically stable and accurate representation of real grasping scenario has been defined. A finger force of 35 N has been applied (Fig. 12b). The value represents a typical power grasp scenario, where the object is firmly grasped for transferring of high loads and moments and to increase stability^36^. Both structures were meshed using quadratic solid FE with 1 mm in size, which was determined based on mesh converged study of 0.25, 0.5 and 1 mm element size.

#### Results of biomechanical simulations

**Figure 13:**
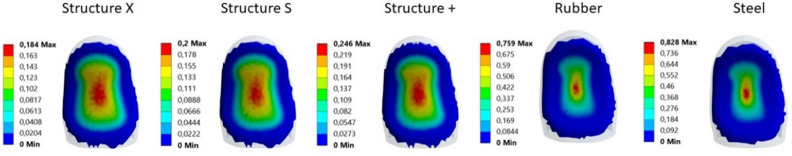
Contact pressure distribution [MPa] at skin-handle interface.

**Figure 14:**
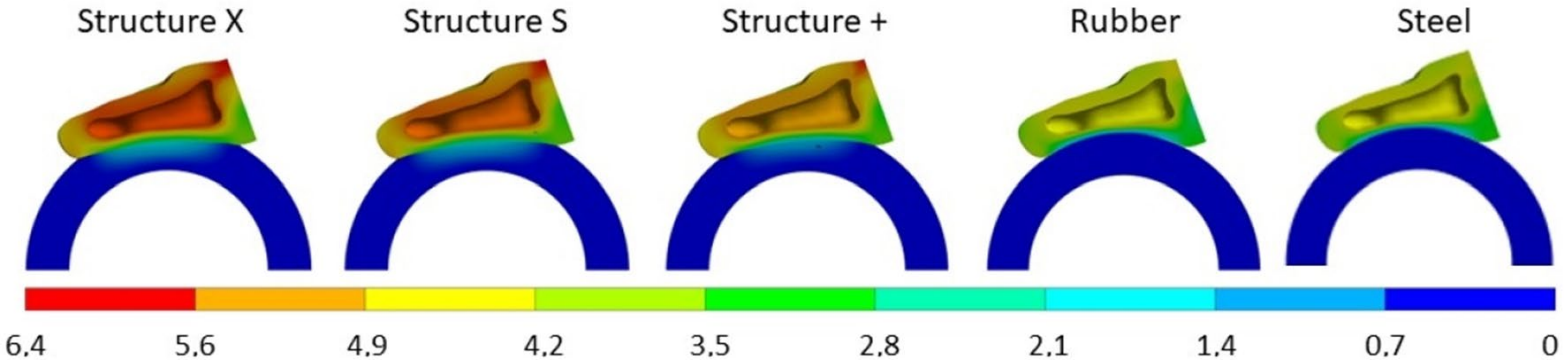
Vertical displacement [mm] at lateral cross-section.

**Figure 15:**
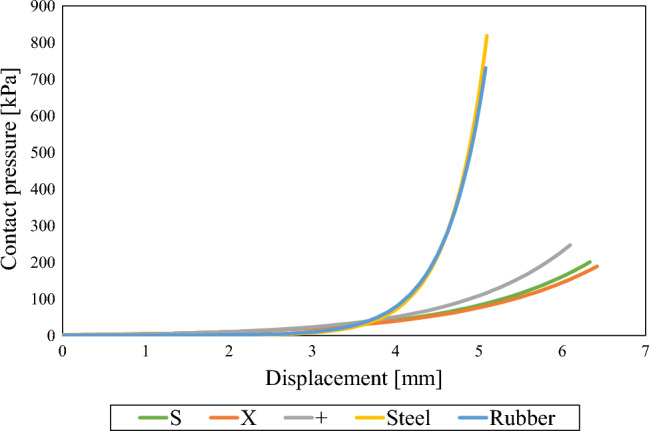
Comparison of contact pressure vs. vertical displacement of the finger and handlebar grip material.

To investigate the effect of the material properties numerical tests were performed according to the mentioned boundary condition for each material and results in terms of contact pressure distribution were extracted (Fig. 13). Vertical displacement of finger and handlebar grip material was provided in lateral cross-section (Fig. 14). Additionally, continuous peak contact pressure for each material was extracted and compared to the combined vertical displacement of finger and handlebar grip (Fig. 15).

### Discussion

Power grasps, among which is also grasping a bicycle handlebar grip, usually require high grasping forces. This results in high contact pressures, which has been reported to induce acute and cumulative trauma disorder development. Therefore, it is crucial to utilise and design handlebar grip interface materials that reduce the loads on the hand and still maintain stability of the grip in hands. Our previous research has shown that stiffness of a grip has a greater impact on tactile perception and comfort than the size and shape of the grip. Grip with appropriate stiffness can increase subjective comfort rating, as it lowers contact pressure and provides a more uniform distribution, while maintaining stability with a controlled low deformation rate. However, research has also shown that using a deformable material that is too soft can diminish the positive effect on tactile perception and comfort.

Results clearly indicate that rubber grip interface material when compared to steel as quasi-rigid material reduces the maximum contact pressure just slightly. This has been already confirmed by our previous research^29^ hence rubber handle material cannot be used for effective contact pressure reduction. On the other hand, all three proposed cellular grips (S, X, +) show significant reduction in maximum contact pressure values. This can be attributed to the deformation of the distinctive mechanical behaviour of cellular geometry proposed in this study. Contact pressure distribution is in the case of cellular grips more uniformly distributed over larger area hence lowering the maximum contact pressure values (Fig. 13), which has been shown by past research to increase comfort rating^29^. Results also show that all proposed cellular structures reduce the contact pressure to the approximate values of pressure discomfort threshold (PDT), while rubber and steel exceed the threshold by several times, surpassing even the pressure pain threshold (PPT). Hence it can be concluded the proposed cellular structures would result in higher comfort rating when compared to the conventional rigid grips. The deformation of the handles consisting of proposed cellular grips show the handle is deformed only at the area where high contact pressures occur, hence lowering the contact pressure while still maintaining stability of the handle in hands due to the small local handle material deformation (Fig. 14). This is also confirmed when comparing the results in terms of continuous peak contact pressure and combined vertical displacement of finger and handle material (Fig. 15). All three proposed cellular grips show similar mechanical behaviour initially (until combined vertical displacement of 3.6 mm), hence providing same stability as quasi-rigid materials (steel, rubber). Afterwards all three cellular grips lower the maximum contact pressure significantly compared to steel and rubber handle materials with increased vertical displacement of just over 1 mm. Previous research has shown that local deformation of handle grips does not impair the stability^30^. Hence, it can be concluded that with the use of proposed cellular grips stability is still maintained, despite effectively lowering the peak contact pressure.

## Conclusions

The different geometries for bicycle handlebars were successfully developed, fabricated and evaluated in this work using computational simulations and experimental testing. The preliminary parametric computational study reveals that the non-auxetic geometries + and X are much stiffer than the other 9 auxetic ones with the S (tetra chiral) geometry. The number of unit cells of the S structures influences the mechanical response of the handlebar grip in all 4 analysed load cases in the way that increasing the unit cell number provides a decrease in the stiffness. Based on this preliminary study, the 3 geometries were chosen for further analysis and fabrication. The X, +, and S geometries were fabricated and experimentally tested on the specially developed clamps. The experimental results shown that the geometries provide similar response up to 0.7 mm of displacement above which, the + structure provides the stiffest response. This is a consequence of the cell walls oriented directly in the radial direction, while the cell walls are already bent in sinusoidal shape before the loading in the case of S structure. The experimental results were then used for the validation of discrete and homogenised computational models. The discrete computational model provides the possibility to precisely analyse the mechanical and deformation behaviour of the handlebar grips and provide also possibilities for further optimisation studies. On the other hand, the homogenised computational model, which provides very good correlation between the experimental and computational results, was used for biomechanical analysis. Biomechanical computational analysis of the proposed cellular grips showed significant reduction in maximum contact pressure despite small additional deformation of the material. Hence, proposed cellular handlebar grips reduce the high contact pressures, but still provide similar stability and hereby improve the handlebar ergonomics.

## Data Availability

The raw/processed data supporting the findings of this study are available from the corresponding authors on request.
